# Small Molecules, Inhibitors of DNA-PK, Targeting DNA Repair, and Beyond

**DOI:** 10.3389/fphar.2013.00005

**Published:** 2013-01-31

**Authors:** David Davidson, Lilian Amrein, Lawrence Panasci, Raquel Aloyz

**Affiliations:** ^1^Department of Oncology, Segal Cancer Centre, Lady Davis Institute for Medical Research, Jewish General Hospital, McGill UniversityMontreal, QC, Canada

**Keywords:** DNA-PK, DNA-PKcs, DNA damage, DNA repair, DNA-PK inhibitors

## Abstract

Many current chemotherapies function by damaging genomic DNA in rapidly dividing cells ultimately leading to cell death. This therapeutic approach differentially targets cancer cells that generally display rapid cell division compared to normal tissue cells. However, although these treatments are initially effective in arresting tumor growth and reducing tumor burden, resistance and disease progression eventually occur. A major mechanism underlying this resistance is increased levels of cellular DNA repair. Most cells have complex mechanisms in place to repair DNA damage that occurs due to environmental exposures or normal metabolic processes. These systems, initially overwhelmed when faced with chemotherapy induced DNA damage, become more efficient under constant selective pressure and as a result chemotherapies become less effective. Thus, inhibiting DNA repair pathways using target specific small molecule inhibitors may overcome cellular resistance to DNA damaging chemotherapies. Non-homologous end joining a major mechanism for the repair of double-strand breaks (DSB) in DNA is regulated in part by the serine/threonine kinase, DNA dependent protein kinase (DNA-PK). The DNA-PK holoenzyme acts as a scaffold protein tethering broken DNA ends and recruiting other repair molecules. It also has enzymatic activity that may be involved in DNA damage signaling. Because of its’ central role in repair of DSBs, DNA-PK has been the focus of a number of small molecule studies. In these studies specific DNA-PK inhibitors have shown efficacy in synergizing chemotherapies *in vitro*. However, compounds currently known to specifically inhibit DNA-PK are limited by poor pharmacokinetics: these compounds have poor solubility and have high metabolic lability *in vivo* leading to short serum half-lives. Future improvement in DNA-PK inhibition will likely be achieved by designing new molecules based on the recently reported crystallographic structure of DNA-PK. Computer based drug design will not only assist in identifying novel functional moieties to replace the metabolically labile morpholino group but will also facilitate the design of molecules to target the DNA-PKcs/Ku80 interface or one of the autophosphorylation sites.

## Introduction

Many first-line cancer therapies employ DNA damaging chemotherapeutic agents alone or in combination with ionizing radiation (IR; Seymour et al., 2007; Cardoso et al., 2009; Tol et al., 2009; Raftery and Goldberg, 2010). These therapies primarily target genomic DNA resulting in the formation of highly toxic double-strand DNA breaks (DSBs) leading to cell cycle arrest and cell death. Problematic to cancer treatment, cells have multiple mechanisms for repairing DSBs including homologous recombination (HR; Johnson and Jasin, 2001; Kao et al., 2005; Sung and Klein, 2006) and non-homologous end joining (NHEJ; Burma et al., 2006; Lieber et al., 2006; Bennardo et al., 2008; Lieber, 2008). HR, an error free repair process, depends on the availability of a homologous DNA template and, as such, functions primarily in the G2/M phase of the cell cycle when sister chromatids are in close proximity. NHEJ, although error prone compared to HR, is thought to be the main pathway for the repair of DNA DSBs. This pathway is able to function at all stages of the cell cycle and does not require the presence of a homologous template. The risk of mutation to an essential gene as a result of NHEJ is balanced by the benefit of avoiding catastrophic cell division in the presence of DSBs. As such, it has been hypothesized that targeting the molecular machinery driving the DNA damage response (DDR), particularly NHEJ and DSB repair, with small molecule inhibitors, will effectively enhance the efficacy of current cancer treatments that generate DNA damage.

## DNA Dependent Protein Kinase

One such target, DNA dependent protein kinase (DNA-PK), a nuclear serine/threonine kinase, is a critical protein facilitating NHEJ (Durocher and Jackson, 2001; Yang et al., 2003, 2009; Collis et al., 2005; Dobbs et al., 2010; Neal and Meek, 2011; Neal et al., 2011). The DNA-PK holoenzyme is composed a regulatory heterodimer (Ku70 and Ku80 subunits) and a 460 kD catalytic component, DNA-PKcs. Based on amino acid sequence homology DNA-PK is a member of the phosphatidylinositol-3-kinase superfamily, however, since it possesses no apparent lipid kinase activity it has been classified along with Ataxia Telangiectasia Mutated (ATM), Ataxia Telangiectasia and Rad3 Related (ATR), Mammalian Target of Rapamycin (m-Tor), and Suppressor of Morphogenesis in Genitalia-1 (SMG-1) as part of the phosphatidyl inositol 3-kinase-like (PIKK) protein kinase group (Durocher and Jackson, 2001). Along with ATM and ATR, DNA-PK has a central function in the DSB response and collectively these three kinases are capable of phosphorylating upwards of 700 proteins (*in vitro*; Callen et al., 2009). DNA-PK itself is a target of ATM and ATR and is essential to NHEJ, V(D)J recombination and class switch recombination (Ma et al., 2002; Callen et al., 2009; Neal and Meek, 2011), all of which require the repair of DSBs. *In vitro* DNA-PKcs is activated by free DNA ends and is capable of phosphorylating several protein substrates including the Ku70/80 heterodimer, histone variant H2AX, replication associated protein A (RPA), and autophosphorylation (Stiff et al., 2004; Reitsema et al., 2005; Wang et al., 2005; Mukherjee et al., 2006; Koike et al., 2008; An et al., 2010). Furthermore, DNA-PK depended phosphorylation of S473 of AKT in response to platinum based chemotherapy has been shown to inhibit apoptotic response limiting drug efficacy (Stronack et al., 2011). There is also *in vitro* evidence that DNA-PK interacts with or influences p53 and p21 activities leading to cellular senescence and apoptosis (Azad et al., 2011; Rudolf et al., 2011; Stronack et al., 2011; Wei et al., 2012). Even so, only a few *in vivo* DNA-PK substrates are known, these include DNA-PKcs autophosphorylation and histone variant H2AX. H2AX phosphorylation on S139 (γH2AX) has been linked to Akt activity downstream of DNA-PK with S473 of Akt being phosphorylated by DNA-PK. In response to this phosphorylation GSK3β, a negative regulator of DNA-PK, is phosphorylated on Ser9 which prevents GSK3β from dephosphorylating γH2AX (An et al., 2010). Despite the small number of substrates identified *in vivo* DNA-PK kinase activity is essential to efficient DSB repair as only wild type and not kinase dead enzyme can rescue IR sensitivity in DNA-PKcs defective V3 cells (Chen et al., 2005). Furthermore, cells displaying resistance to DNA damaging agents had increased levels of DNA-PKcs activity and cells deficient in DNA-PKcs had enhanced sensitivity to DNA damaging agents (Soubeyrand et al., 2003; Shinohara et al., 2005; Morris et al., 2011). Also, although phosphorylation of DNA-PK and DNA-PK kinase activity are not required for initial recruitment to DNA damage sites they are required for efficient repair of DSBs (Davis et al., 2010). It has been proposed that DNA-PK functions as a scaffolding protein to align the broken DNA ends and assist in the localization of repair factors. It may also play a regulatory role by physically blocking unrepaired DNA ends preventing their degradation, then, through autophosphorylation, releasing them for repair once proper alignment has been achieved (Neal and Meek, 2011; Neal et al., 2011).

Of note, a recent review by Kong et al. (2011) discusses physiological functions of DNA-PK beyond its’ role in DNA repair.

These authors note that the abundant expression of the large DNA-PK protein molecule in numerous cell types does not impart improved DNA repair abilities, suggesting other important functions for this molecule may exist. Evidence presented suggests that DNA-PK has important roles in regulating gene response to feeding/insulin stimulation. Furthermore, it is suggested that DNA-PK has an active part in the regulation of homeostasis of cell proliferation. Given that cancer cells generally have a high rate of proliferation and have a high rate of metabolic activity these additional functions suggest further mechanisms by which DNA-PK inhibition can target cancer cells.

## Mechanism

Regarding the role of DNA-PK in DNA repair a recent model of NHEJ was proposed by Neal and Meek (2011) that suggests DSB repair is initiated when the regulatory component of DNA-PK, Ku70/Ku80, comes in contact with DNA ends at DSB sites. The Ku heterodimer, which is able to bind to various double-stranded end structures (blunt ends, 3′ or 5′ overhangs, covalently closed hairpins) in a sequence independent manner, forms an asymmetric ring that encircles double-stranded DNA ends. Subsequently, DNA-PKcs is recruited and binds to the C-terminal portion of Ku80 (Morris et al., 2011). DNA bound Ku then translocates inwards away from the broken end significantly stimulating the kinase activity of DNA-PKcs. It is proposed that homo-dimeric interaction of DNA bound DNA-PK complexes brings corresponding broken ends into close proximity and subsequently acts as a scaffold for recruiting downstream repair proteins such as XRCC4, DNA ligase IV, Artemis, XLF, and aprataxin. Activity of this larger protein complex ultimately results in the repair of DSBs (Kuo et al., 2006; Mansour et al., 2008). Molecular models of DNA-PK associated DSB repair have been reviewed in detail by Thompson (2012). The initial step of DSB repair occurs rapidly with both ATM and DNA-PK being recruited to DNA damage sites. DNA-PK is recruited within 60 s and recruitment peaks after 10 min. In contrast ATM recruitment peaks after 20 min. DNA-PK rapidly dissociates after reaching peak levels and in contrast ATM lingers for a prolonged period. Dissociation of DNA-PK, ATM, and other DNA repair factors from DSB sites is associated with the resolution of γH2AX foci suggesting successful DSB repair. In contrast, DNA-PK showed persistent localization in NHEJ deficient cells (Uematsu et al., 2007). These data suggest a model where DNA-PK and ATM hold separate and distinct functions in DSB repair with DNA-PK functioning as a scaffold and ATM acting as a signaling kinase.

## Phosphorylation

DNA dependent protein kinase is clearly important for efficient repair of DSBs and to date as many as 40 potential phosphorylation sites have been identified on DNA-PK with the majority of these being located in two major clusters: (1) the ABCDE cluster containing S2612, S2624, T2609, T2620, T2638, T2647 and 2) the PQR cluster containing sites S2023, S2029, S2041, S2053, and S2056 (Morris et al., 2011; Neal and Meek, 2011; Neal et al., 2011). Mechanistically, experimental evidence suggests that unphosphorylated DNA-PK blocks DNA ends inhibiting efficient ligation and that autophosphorylation generates a large conformational change opening the structure of DNA-PK (Davis et al., 2010; Morris et al., 2011) releasing it from aligned DNA ends. For example, cells expressing mutations at S2056 or T2609 have tight binding of DNA-PK to DNA DSBs and a slow off rate at sites of DNA damage (Davis et al., 2010). The structure of the DNA-PKcs molecule has three major domains facilitating this functionality: a kinase domain, a large N-terminal domain that includes two long arms into which DNA is proposed to thread and a DNA binding pocket. The phosphorylations of amino acids from the respective clusters, S2056 (PQR) and T2609 (ABCDE), are likely distinctly regulated events as the physical locations of these residues are far apart (Chen et al., 2005). Furthermore, phosphorylations of these clusters may be functionally distinct in that ABCDE and PQR clusters were shown to regulate DNA end processing and DNA repair pathway choice in a reciprocal manner. For example, blocking at the amino acid cluster ABCDE inhibited end processing and HR while blocking at the PQR cluster enhanced these processes. Additionally, it was observed that S2056 phosphorylation increased incrementally and reached maximum levels at 50 Gy of IR. This was in contrast to T2609 whose phosphorylation reached maximum levels at 10 Gy. Based on the use of ATM and DNA-PK knockout cell lines and DNA-PK and ATM specific siRNAs, it was proposed that the S2056 phosphorylation is an autophosphorylation event that occurs in trans while the T2609 phosphorylation is at least in part due to ATM activity (Collis et al., 2005). Further examining the role of autophosphorylation, Neal and Meek (Neal and Meek, 2011; Neal et al., 2011) noted that DNA-PK a relatively abundant protein inhibits the process of HR in a titratable manner. They also noted that this inhibition is absolutely dependent on its’ enzymatic activity and is distinctly modulated by phosphorylation. Through extensive examination of various phosphorylation sites they observed that some sites impact NHEJ negatively while promoting HR. They concluded that failed NHEJ results in increased HR and conversely that in most cases proficient NHEJ inhibits HR. In summary current data suggests that DNA-PK has two important phosphorylation sites: (1) a cluster containing S2056 that is primarily autophosphorylated and (2) a cluster containing sites T2609 and T2647 that are at least in part phosphorylated by ATM. Phosphorylation at these sites is critical as its absence results in retention of DNA-PK foci at damage sites for longer periods. All of these together illustrate the potential for blocking DNA repair by inhibiting DNA-PK phosphorylation. Thus there is vast potential for the use of DNA-PK inhibitors in enhancing DNA damaging chemotherapies.

## DNA-PK Inhibitors

**Figure 1:**
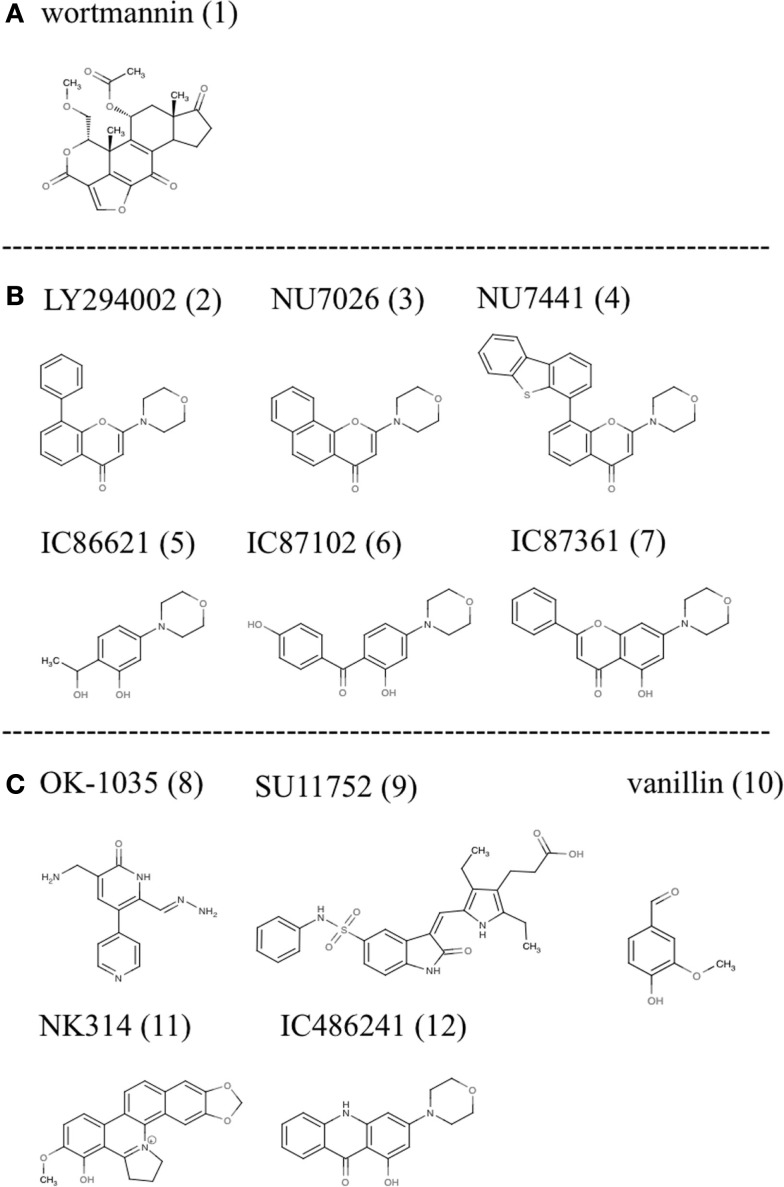
**Small molecule inhibitors of DNA-PK: (A) wortmannin, (B) LY294002 derivatives, and (C) other molecules with demonstrated inhibitory activity**.

As such, to date the most successful approach to DNA-PK inhibition has been with small molecules that target the ATP binding site of the kinase domain (Allen et al., 2003). Various investigators using compound libraries have identified a small group of molecules that effectively inhibit DNA-PK kinase activity (Figure 1). However, despite their specificity and potency these molecules have been hampered by poor pharmacokinetic properties.

One of the first identified inhibitors, wortmannin (1), a furanosteroid metabolite of the fungi *Penicillium funiculosum*, has been used experimentally to inhibit DNA-PK. It is a non-competitive general inhibitor of PI-3 kinases and has an IC_50_ in the nanomolar range (5 nM for PI3K and equipotent for DNA-PK). Its’ mode of action involves the irreversible alkylation of lysine 802, an amino acid residing in the active site of DNA-PKcs that is critical for the phosphate transfer reaction. Experimentally wortmannin has proven to be an effective radiosensitizer with a dose reduction factor (DRF) for IR at 10% survival between 1.4 and 3. Despite its’ experimental efficacy: lack of specificity, poor solubility in aqueous solution, and *in vivo* toxicity limit clinical application of this compound (Collis et al., 2005).

LY294002 (2), a morpholine derivative of the plant flavonoid quercetin, is also a non-specific DNA-PK inhibitor used in many studies (Vlahos et al., 1994; Sauveur-Michel et al., 2009). It is a competitive inhibitor that binds reversibly to the kinase domain of DNA-PK with an IC_50_ of 1.4 μM producing a DRF at 10% survival with IR of 1.5–1.8 (Collis et al., 2005). Rapid metabolic clearance (1 h), *in vivo* toxicity, and lack of specificity make clinical evaluation unfeasible in humans. However, LY294002 has proven to be a productive lead compound and biochemical modifications have produced a series of compounds with more favorable properties (Fuhrman et al., 2008; Clapham et al., 2011). These modifications using LY294002 as template have improved specificity with regards to DNA-PK inhibition (Ihmaid et al., 2011).

One such compound NU7026 (3) was over 50 fold more selective for DNA-PK than other PI-3Ks including ATM and ATR. NU7026 had an IC_50_ of 0.23 μM for DNA-PK, 13 μM for PI3K, and >100 μM for ATM or ATR (Peddi et al., 2010). Furthermore, this drug potentiated the growth inhibition of a variety of chemotherapeutic agents including: idarubicin, daunorubicin, doxorubicin, etoposide, amsacrine, mitoxantrone but did not potentiate camptothecin or cytosine arabinoside (Willmore et al., 2004). Cell based assays showed a DRF for this drug with IR at 10% survival of mouse embryonic fibroblasts of 1.5 (Nutley et al., 2005). However, metabolic lability remains problematic as monohydroxylation occurs on position 2 of the morpholino group resulting in opening of the morpholino ring. Thus it is not likely that sufficiently high concentrations of this drug can be obtained in patients over the 4 h period required to synergize radiation and or chemotherapy. Furthermore, poor solubility in saline solutions limits the use of osmotic mini pumps. It has been suggested that the 2-morpholin-4-yl substituent is required for inhibitory activity. As such attempts have been made to maintain this structure while modifying other parts of the molecule to improve pharmacokinetics. Problematically the core ring structure is not conducive to chemical modifications. As such, Hollick et al. proposed modification of pyran-4-ones and thiopyran-4-ones and obtained suitably potent and selective DNA-PK inhibitors. Although this approach obtained novel compounds screening continues to identify compounds with improved biological and pharmaceutical properties (Hollick et al., 2003).

NU7441 (4) another drug based on the LY294002 backbone with improved potency showed similar promise to NU7026. In cell lines it showed strong inhibition of DNA-PK with an IC_50_ of 0.3 μM for DNA-PK and 7 μM for PI3K. In the presence of this compound doxorubicin and IR induced DSBs persisted for prolonged periods and HR activity increased modestly (Rad51 foci; Tavecchio et al., 2012). Zhao and Curtin (Zhao et al., 2006) noted that NU7441 increased the cytotoxicity of IR and etoposide in SW620, LoVo, and V3-YAC colon cancer cells but not in V3 cells indicating that potentiation of DNA damage and cell death was largely due to DNA-PK inhibition. Furthermore, NU7441 substantially delayed the repair of IR and etoposide induced DSBs and appreciably increased G2/M accumulation associated with IR, etoposide and doxorubicin in SW620, and LoVo colon cancer cells. In xenograft colon cancer models NU7441 concentrations in the tumor necessary for chemopotentiation *in vitro* were maintained for at least 4 h at non-toxic doses, a significant improvement over NU7026. Additionally, etoposide induced tumor growth delay was increased two-fold without enhancing toxicity. Thus experiments with NU7441 have shown that pharmacokinetics of these inhibitors can be improved but their marginal effects on radiation and chemotherapy have halted further development.

Other compounds based on the LY294002 structure have been tested including IC86621 (5), IC87102 (6), and IC87361 (7). In cell culture these recapitulate the phenotype of DNA-PK defective cell lines including those from SCID mice and directly inhibit the repair of DNA DSBs and enhance the cytotoxicity of physical and chemical agents that induce DSB formation. As with other compounds tested IC86621 showed increased sensitization to IR and decreased repair of spontaneous and IR induced DSBs. Since, these compounds are non-toxic there main therapeutic potential is to enhance the effects of DNA damaging therapies on cancer cells (Kashishian et al., 2003). DNA-PKcs was strongly inhibited and even at concentrations of 100 μM, IC86621 had no activity against the closely related protein kinases ATM, ATR, or FRAP. Additionally, IC87361 showed significant radiosensitization at 6 Gy and significant growth delay in xenograft models. At 10 μM concentrations this drug had no intrinsic growth inhibitory properties but significantly potentiated the effect of radiation in ovarian cancer cells. It was observed that at least 4 h exposure to a 10 μM concentration of this drug was required for radiosensitization and that 24 h of exposure further improved this effect. This time of drug exposure is likely to be important for positive therapeutic effects and illustrates that pharmacokinetic properties such as: rapid clearance from circulation and low bioavailability (Shinohara et al., 2005) are the major barriers to the clinical use of these compounds.

Other compounds based on different chemical structures and found to have inhibitory activity against DNA-PK include OK-1035 (8) and SU11752 (9). OK-1035 with an IC_50_ for DNA-PK of 100 μM is unlikely to be a useful drug *in vivo* (Take et al., 1996). Similarly, SU11752 was shown to lack the required potency for *in vivo* studies.

The phenolic aldehyde vanillin (10), derived from some species of vanilla pods, has also been shown to inhibit DNA-PK in cell free extracts. This inhibition was shown to be specific to NHEJ and selective for DNA-PK over ATM and ATR with a DRF of 1.3 at 10% survival following IR of human ovarian carcinoma cells (Durant and Karran, 2003).

Recently the anticancer agent NK314 (11), possessing activity against topoisomerase II alpha (TIIα), was shown to induce the degradation of DNA-PKcs resulting in impaired DNA DSB repair. In other words NK314 appears to be an inhibitor with dual specificities for TIIα and DNA-PKcs with potential activity against adult T-cell leukemia-lymphoma (ATL; Hisatomi et al., 2011). Although the mechanism by which this drug inhibits DNA-PK remains unclear it illustrates the potential for combining DNA-PK activity with chemotherapeutics. The combination is in this case all the more effective because the two activities are delivered as a single reagent. This drug is currently in clinical trials for the treatment of ATL illustrating the potential for using DNA-PK inhibitors in the clinic.

Recent studies in our laboratory have explored the efficacy of acridone based compounds as DNA-PK inhibitors (Davidson et al., 2012a,b). These studies sought to measure the degree of synergy induced by the specific small molecule inhibitor of DNA-PK, IC486241 (ICC) (12), with irinotecan (SN38) or oxaliplatin. Significant reductions in the IC_50_ values of SN38 were observed at 5 and 10 μM of DNA-PK inhibitors. Moreover, at 1–2 μM (attainable concentrations with ICC in mice), these DNA-PKcs inhibitors demonstrated synergistic reductions in the IC_50_ of SN38. Flow cytometric data indicated that SN38 and SN38 in combination with DNA-PKcs inhibitors showed dramatic G2/M arrest at 24 h. Furthermore, reduced phosphorylation of DNA-PKcs and increased DNA damage were observed at this time point with SN38 in combination with DNA-PKcs inhibitors as compared to cells treated with SN38 alone. SN38 alone and in the presence of ICC increased nuclear Rad51 protein levels. Furthermore, inhibition of DNA-PKcs increased HRR suggesting that NHEJ is a negative regulator of HRR. This observation was corroborated by Neal et al. (2011), who showed that DNA-PK enzymatic activity could inhibit HRR in a titratable fashion. In addition these authors also showed that some phosphorylations of DNA-PK had the opposite effect, that is to say, inhibition of NHEJ and promotion of HRR. Of interest, Amrein et al. (2011) observed a similar relationship between the HRR and NHEJ pathways in chronic lymphocytic leukemia (CLL) lymphocytes treated with chlorambucil (CLB). Imatinib and Nilotinib, two potent inhibitors of the non-receptor tyrosine kinase c-abl, caused a reduction in HRR and a corresponding increase in DNA-PK (NHEJ) activity. Together these observations suggest that DNA-PK has a significant function in choosing the pathway of DNA repair. A similar series of experiments showed the degree to which ICC synergizes the cytotoxicity of DNA damaging agents in three genetically diverse breast cancer cell lines. In this study, improved cytotoxicity and significant synergy were observed with both anticancer agents in the presence of non-toxic concentrations of ICC. Moreover, ICC decreased doxorubicin-induced DNA-PKcs autophosphorylation on Ser2056 and increased doxorubicin-induced DNA fragmentation. Thus, the novel DNA-PKcs inhibitor, ICC, synergistically sensitized 3 breast cancer cell lines to doxorubicin and cisplatin. Enhanced efficacy of doxorubicin was achieved by inhibiting NHEJ resulting in increased accumulation of DNA damage. Pharmacokinetic studies of these compounds in mice showed maximum serum concentrations of 4.6 μM 1 min post intravenous injection and terminal serum half-lives of 23.8 min (personal communication, Luipold pharmaceuticals). Thus although this drug shows promising synergy with chemotherapeutics in cell culture, future use will depend on developing compounds with improved solubility and serum half-life.

## Nucleotide and Antibody Inhibitors

Although the majority of studies have focused on the use of small organic compounds to inhibit DNA-PK, a few have focused on nucleotide and antibody based inhibitors and shown that these may also have clinical efficacy in the inhibition of DNA-PK. In fact due to the biological nature of these molecules they may overcome the two primary obstacles faced by small organic compounds known to inhibit DNA-PK, namely: poor solubility’s and short serum half-lives. GRN163L (Imetelstat; GRN), a 13-mer oligonucleotide developed as a direct inhibitor of the telomerase active site illustrates how nucleotides can influence DNA-PK function. In a recent study in CLL lymphocytes this molecule was shown to inhibit DNA-PK phosphorylation and increase H2AX phosphorylation in response to treatment with the nucleotide analog fludarabine (flu; Shawi et al., 2011). The authors observed that activity was correlated with low expression levels of the DNA-PK subunit Ku80 and that GRN inhibited DNA-PK phosphorylation at a level equivalent to NU7026. Together these observations indicate that GRN in addition to inhibiting telomerase activity enhances chemotherapy by inhibiting DNA-PK activity and repair of DNA damage. It would be of significant interest to know if GRN synergizes the killing of other cancer types in the presence of a variety of DNA damaging therapies.

In addition to oligonucleotides, antibody based inhibitors may also have a role to play in DNA-PK inhibition. Antibodies, although effective in the inhibition of cell surface targets (Scott, 2012) are typically ineffective against intracellular targets because of their inability to penetrate the plasma membrane (Cardinale and Biocca, 2008). However, recent work in which the DNA-PK specific single-chain antibody variable region fragment ScFv 18-2 was linked to folate via a scissile disulfide linker showed efficacy in delivering the antibody fragment to cell nuclei via folate mediated endocytosis. This product resulted in decreased DNA-PK phosphorylation, increased H2AX phosphorylation, and radiosensitization (Xiong et al., 2011). This work is of interested for a number of reasons. Firstly, it illustrates the potential of antibody based therapies that target intracellular molecules such as DNA-PK and secondly it illustrates the efficacy of combi-molecules composed of a cell surface receptor substrate and a chemotherapeutic agent. The receptor substrate as in this case can facilitate transit of the plasma and nuclear membranes but may also be used to target drugs to cancers over-expressing specific surface receptors.

In conclusion DNA-PK inhibition using small molecule inhibitors holds promise for improving cancer therapy. However, molecules currently known to specifically inhibit DNA-PK are limited by poor pharmacokinetics: metabolic lability leads to a short serum half-life. Future improvement in DNA-PK inhibition will likely be achieved by designing new molecules based on the recently reported crystallographic structure of DNA-PK (Sibanda et al., 2010; Ihmaid et al., 2012). Computer based drug design will not only assist in identifying novel functional moieties to replace the metabolically labile morpholino group but will also facilitate the design of molecules to target the DNA-PKcs/Ku80 interface or one of the autophosphorylation sites. Of significant interest novel approaches using DNA-PK specific antibodies or oligonucleotides may also be of great value as they may overcome the fundamental weaknesses of currently available small molecule inhibitors of DNA-PK, namely: poor solubility and short serum half-life. To date the latter have only been tested against DNA-PK *in vitro*, future work will demonstrate if these approaches have significant *in vivo* efficacy.

## Conflict of Interest Statement

The authors declare that the research was conducted in the absence of any commercial or financial relationships that could be construed as a potential conflict of interest.
